# Comparative Clinicopathologic Characteristics and Outcomes of Paediatric and Adult Xp11 Translocation Renal Cell Carcinomas: a Retrospective Multicentre Study in China

**DOI:** 10.1038/s41598-020-59162-5

**Published:** 2020-02-10

**Authors:** Wenliang Ma, Ning Liu, Wenyuan Zhuang, Weijian Li, Feng Qu, Jing Sun, Wei Xu, Lihua Zhang, Ruipeng Jia, Linfeng Xu, Xiaozhi Zhao, Xiaogong Li, Gutian Zhang, Hongqian Guo, Dongmei Li, Weidong Gan

**Affiliations:** 1 Department of Urology, Drum Tower Clinical Medical School of Nanjing Medical University, Nanjing, Jiangsu China; 2 Department of Urology, Nanjing Drum Tower Hospital, The Affiliated Hospital of Nanjing University Medical School, Nanjing, Jiangsu China; 3 Department of Oncology, Jiangsu Province Hospital, The First Affiliated Hospital of Nanjing Medical University, Nanjing, Jiangsu China; 4 Department of Pathology, Jiangsu Cancer Hospital, The Affiliated Cancer Hospital of Nanjing Medical University, Nanjing, Jiangsu China; 5 Department of Pathology, Zhongda Hospital Southeast University, Nanjing, Jiangsu China; 6 Department of Urology, Nanjing First Hospital, The Affiliated Nanjing Hospital of Nanjing Medical University, Nanjing, Jiangsu China; 7 Immunology and Reproduction Biology Laboratory & State Key Laboratory of Analytical Chemistry for Life Science, Medical School, Nanjing University, Nanjing, Jiangsu China; 8 Jiangsu Key Laboratory of Molecular Medicine, Nanjing University, Nanjing, Jiangsu China

**Keywords:** Cancer epigenetics, Renal cell carcinoma

## Abstract

This study aimed to compare the clinicopathologic features and prognosis in patients with Xp11 translocation renal cell carcinomas (RCCs). In total, 8083 RCCs were screened at five centres from January 2007 to December 2018, including 8001 adults (≥18 years) and 82 children (<18 years). Finally, 73 adults and 17 children were identified as Xp11 translocation RCCs, accounting for 1.1% (90 of 8083) of the RCCs. However, 4 children and 1 adult were excluded because of loss to follow-up when performing the survival analysis. The proportion of paediatric and adult Xp11 translocation RCCs was 20.7% (17 of 82) and 0.9% (73 of 8001) of RCCs, respectively, and the incidence in children and adults was significantly different (*P* < 0.01). Lymph node positivity (LN+) most commonly occurred in children (58.8%) compared with adults (28.8%; *P* = 0.02), but children with LN+ showed significantly higher five-year overall survival and progression-free rates (OS: 75.0%; PFS: 64.8%) than adult patients (OS: 40.3%; PFS: 0%) (log-rank *P*^PFS^ < 0.01; *P*^OS^ = 0.04). Multivariable analysis indicated that local lymph node metastasis was associated with both PFS (HR = 0.10; 95% CI 0.02–0.51; *P* = 0.01) and OS (HR = 0.11; 95% CI 0.01–0.98; *P* = 0.04) in adults. Adult patients with LN+ may indicate a worse prognosis than paediatric patients.

## Introduction

Xp11 translocation renal cell carcinomas (RCCs) are characterized by several different chromosomal translocations involving Xp11 and the formation of TFE3 fusion genes, followed by overexpression of TFE3 protein^1^. The first case was a 2.4-year-old child diagnosed by karyotype analysis in 1986^2^. In 2004, Xp11 translocation RCCs were classified as a distinct entity of RCC by the World Health Organization (WHO)^3^. Recent reports have found that RCC associated with t(6; 11) (p2l; q12)/TFEB gene fusions is exceedingly similar to Xp11 translocation RCCs with respect to clinical characteristics, pathology, and molecular genetics^4^. Given that both TFE3 and TFEB pertain to the microphthalmia-associated transcription factor family, Xp11 translocation RCCs and RCC associated with t (6; 11) (p2l; q12)/TFEB gene fusions were reclassified as MiT family translocation RCCs in the 2016 WHO renal tumour classification scheme^5^.

TFE3 protein immunohistochemistry (IHC) combined with the fluorescence *in situ* hybridization (FISH) assay was an effective method for the diagnosis of Xp11 translocation RCCs^6^. Several clinical studies have shown that the incidence of paediatric Xp11 translocation RCCs is significantly higher than that of adults^7–9^. Because its histomorphology is extremely similar to clear cell renal cell carcinoma and papillary renal cell carcinoma, it is easily misdiagnosed and missed. Therefore, the actual incidence of the disease is worthy of further investigations with a large sample size. Patients with Xp11 translocation RCCs often present at advanced stages^10^, but paediatric patients with a higher incidence of regional lymph node positivity (LN+) seem to have a better prognosis than adults when regional lymph node involvement was completely resected^11,12^. In addition, the natural history of the disease is variable between children and adults based on clinical experience. Meta-analysis showed that clinical characteristics between children and adults were distinctive, including the incidence of lymphatic metastases, tumour stage and prognosis^13^. In view of the lack of a large sample size and longer follow-up for this relatively recent category of tumours, we grouped five urological cancer research institutes together to compare the clinicopathologic features and outcomes in patients with paediatric and adult Xp11 translocation RCCs.

The purpose of the present study was to compare the clinicopathologic features and outcomes of Xp11 translocation RCCs between children and adults by multi-institutional analysis of patients.

## Materials and Methods

### Patient selection

The retrospective study was conducted according to the ethical principles of the Helsinki Declaration II, and written informed consent was obtained from both adult patients and legal guardians of paediatric patients before the clinical investigations were conducted. The present study was approved by the Institutional Review Board of Nanjing Drum Tower Hospital, Jiangsu Province Hospital, Jiangsu Cancer Hospital, Zhongda Hospital Southeast University and Nanjing First Hospital. We have verified that the data collected at five institutions can be treated as independent data (Supplementary Tables S1–S3). We screened 8083 RCCs from patients consecutively treated by surgery at five centres from January 2007 until December 2018 (Table 1), including 8001 adults (≥18 years) and 82 children (<18 years). Eventually, 73 adults and 17 children who were diagnosed with Xp11 translocation RCCs were included in the analyses for whom clinical information was available, but 4 children and 1 adult were excluded from the study because of loss to follow-up when performing the survival analysis. We included patients who were identified by TFE3-IHC combined with the break-apart FISH assay performed on paraffin-embedded tissue blocks^14^. Patients were excluded for incomplete clinical and follow-up data, negative TFE3-IHC and break-apart FISH assays.

**Table 1:** The number of cases per institution is provided.

Institution	Included		Excluded	
	Adults	Children	Adults	Children
Nanjing Drum Tower Hospital	46	6	0	2
Jiangsu Province Hospital	14	1	1	2
Jiangsu Cancer Hospital	7	0	0	0
Zhongda Hospital Southeast University	2	6	0	0
Nanjing First Hospital	3	0	0	0

### Evaluation of clinicopathologic data

The clinicopathologic data for patients in two groups were analysed, including sex, clinical symptoms, operation, laterality, pathological features (pT stage, local lymph node metastasis, vena cava tumour thrombosis, tumour boundary, nuclear grade and American Joint Committee On Cancer [AJCC]) stage), adjuvant therapy and clinical outcomes (stable, recurrent or dead). Symptoms were analysed when patients had one or more initial symptoms. A clear tumour boundary was defined as a complete continuous tumour pseudocapsule between the tumour mass and normal kidney tissue. The 2016 WHO/ISUP pathological nuclear grading system was referenced by the nuclear grade standards. TNM staging was based on the eighth edition issued by the AJCC staging in 2017. All of the patients were followed up until the time of death or loss to follow-up.

### Statistical analysis

Count data were expressed as percentages. Categorical variables were analysed using the Pearson chi-square test, while it was performed using Fisher’s exact test when the expected frequency was less than 5 in any cell. Progression-free survival (PFS) was defined from the initiation of surgery to the date of disease progression or death outcome or censored at last follow-up. Overall survival (OS) was defined as the time interval between the date of surgery and the date of death or last follow-up. The Kaplan-Meier method was used to estimate both PFS and OS, and statistical comparisons were assessed by the log-rank test. A Cox proportional hazards model was used to evaluate the predictive role of the factors that showed significance in the long-rank test. A two-side p-value of <0.05 was considered statistically significant. SPSS software version 23.0 (SPSS Inc., Chicago, IL, USA) was used for all statistical analyses. The survival curves were drawn using GraphPad Prism software version 5.0.

## Results

### Epidemiological characteristics

Of the 8083 patients with RCCs, 90 cases (male to female ratio of 1:1.43) with a median age of 28 years old (ranging from 3 to 71 years) were diagnosed with Xp11 translocation RCCs, including 17 children, 53 patients between 18 and 45, and 20 patients older than 45. The overall proportion of Xp11 translocation RCCs was 1.1% (90 of 8083), and the proportion of patients younger than 18 years and older than 18 years was 20.7% (17 of 82) and 0.9% (73 of 8001) of RCCs, respectively. The difference in the incidence rates between children and adults was statistically significant (*P* < 0.01).

### Comparative clinicopathologic features and prognosis of children and adults

**Figure 1:**
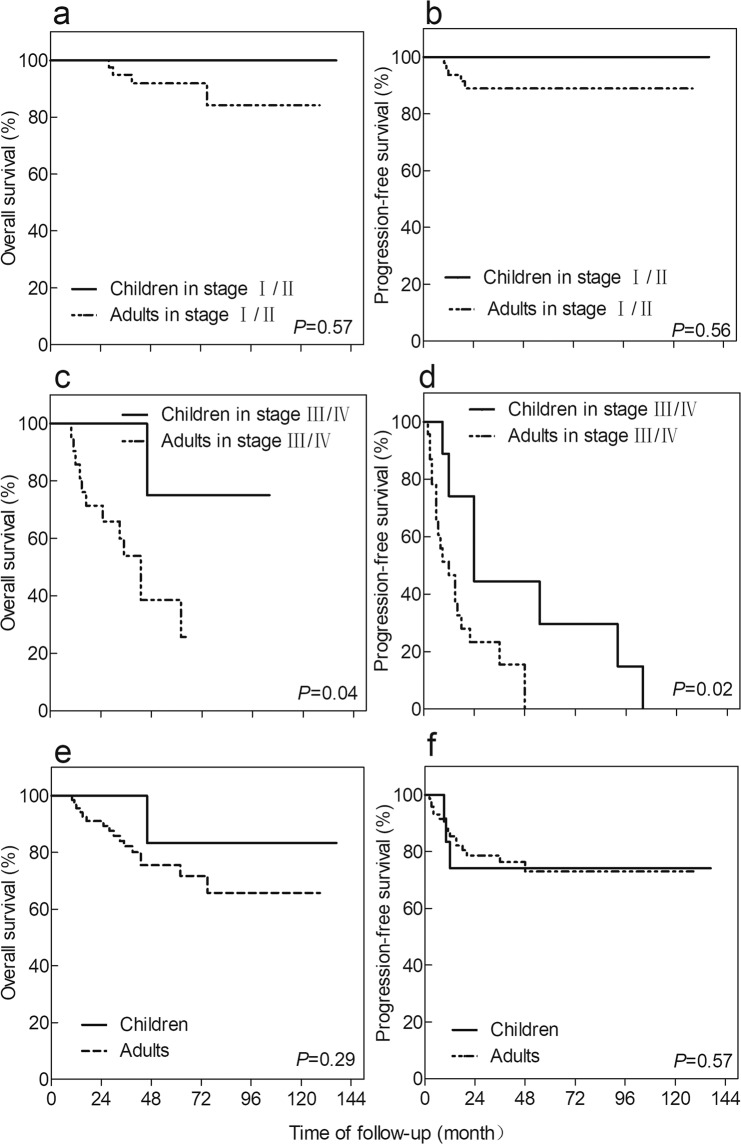
Kaplan-Meier method depicting overall survival (OS) and progression-free survival (PFS) both in all patients and patients grouped by AJCC stage, stratified according to the age of onset. (**a**) Survival analysis of OS in stage I/II patients, (**b**) Survival analysis of PFS in stage I/II patients, (**c**) Survival analysis of OS in stage III/IV patients, (**d**) Survival analysis of PFS in stage III/IV patients, (**e**) Survival analysis of OS in all patients and (**f**) Survival analysis of PFS in all patients.

**Figure 2:**
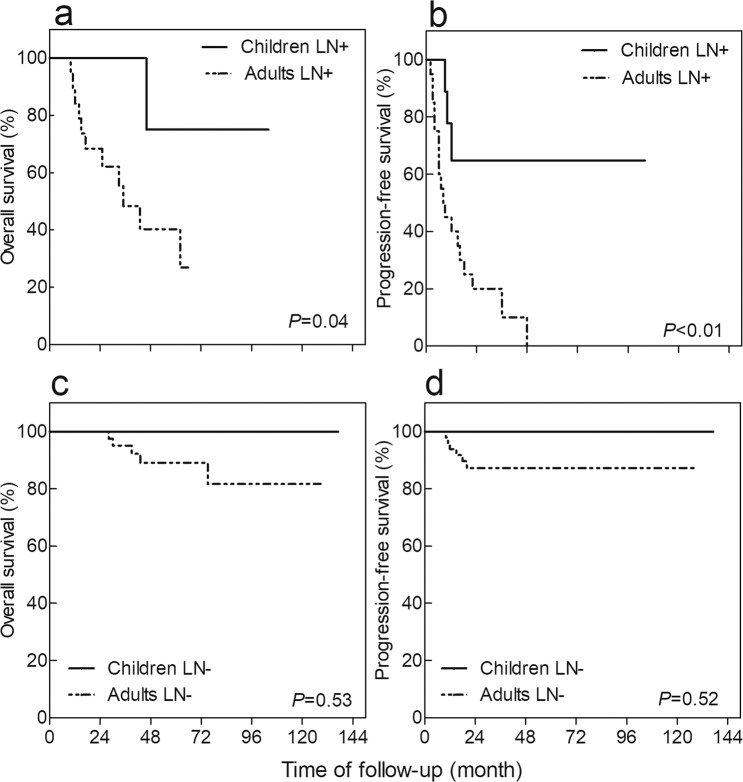
Kaplan-Meier method depicting overall survival (OS) and progression-free survival (PFS) in patients grouped by local lymph node metastasis, stratified according to the age of onset. (**a**) Survival analysis of OS in patients with LN+, (**b**) Survival analysis of PFS in patients with LN+, (**c**) Survival analysis of OS in patients with LN−, and (**d**) Survival analysis of PFS in patients with LN−.

**Figure 3:**
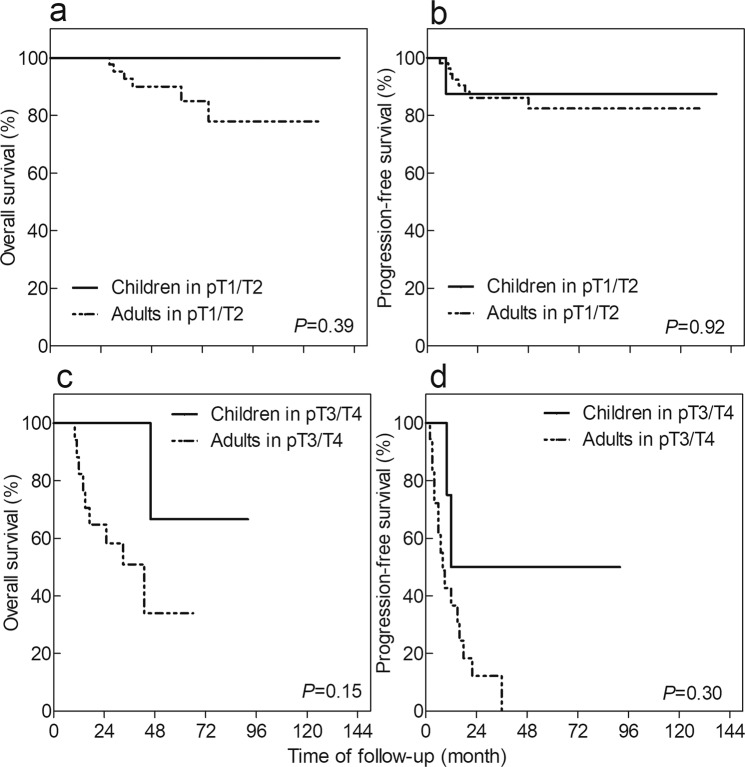
Kaplan-Meier method depicting overall survival (OS) and progression-free survival (PFS) in patients grouped by pT stage, stratified according to the age of onset. (**a**) Survival analysis of OS in pT1/T2 patients, (**b**) Survival analysis of PFS in pT1/T2 patients, (**c**) Survival analysis of OS in pT3/T4 patients, and (**d**) Survival analysis of PFS in pT3/T4 patients.

There were significant differences in the clinicopathologic characteristics and outcomes of the patients within the two groups (Table 2, Figs. 1, 2, 3). The Xp11 translocation RCCs in the adult group were discovered by routine examination (48.7%) or haematuria (28.2%), while those in the paediatric group were diagnosed with haematuria (38.1%) and abdominal mass (23.8%) as the first symptoms. There was a significant difference in the distribution of clinical symptoms between children and adults (*P* < 0.01). Only one child underwent NSS for the primary tumour; however, 24 adult-onset patients received NSS apart from 48 cases of RN (RN: 94.1% children vs 67.1% adults; *P* = 0.03). only one adult patient (1.1%) had sarcomatoid tissue in the tumour, and 10 adult patients (11.1%) had coagulative tumour necrosis. Neither the sarcomatoid histology nor the coagulative necrosis has yet been found in the child patients. Vena cava tumour thrombectomy was performed on 9 cases in the adult group and only one case in the paediatric group. Paediatric patients had a higher five-year overall survival and progression-free rate (OS: 83.3%; PFS: 74.1%) than adult patients (OS: 75.5%; PFS: 63.0%), but they were not statistically significant (log-rank *P*^PFS^ = 0.57; *P*^OS^ = 0.29). Adults showed slightly higher pT stage than children at diagnosis (pT3/T4: 26.0% adults vs 24.5% children; *P* = 0.99), but the prognosis of the two groups at the pT3/T4 stage had no significant difference (log-rank *P*^PFS^ = 0.30; *P*^OS^ = 0.15). Children were more prone to LN+ compared with adults (LN+: 58.8% children vs 28.8% adults; *P* = 0.02), but children with LN+ showed significantly higher five-year overall survival and progression-free survival (OS: 75.0%; PFS: 64.8%) than those of adult patients (OS: 40.3%; PFS: 0%) (log-rank *P*^PFS^ < 0.01; *P*^OS^ = 0.04). There was no significant difference in prognosis between children and adults in stage I/II (log-rank *P*^PFS^ = 0.56; *P*^OS^ = 0.57), but the five-year overall survival and progression-free survival of adults (OS: 38.5%; PFS: 0%) in stage III/IV were lower than those in paediatric patients (OS: 75.0%; PFS: 64.8%) with the same stage (log-rank *P*^PFS^ = 0.02; *P*^OS^ = 0.04). Among child-onset patients in stage III/IV, 2 patients received immunotherapy, and only one child had targeted treatments. However, in the adult group, 26 cases in stage I/II and 5 cases in stage III/IV received immunotherapy, and 13 cases in stage III/IV and the 5 recurrence cases received targeted therapy.

**Table 2:** Clinical and pathological characteristics of 90 patients.

Characteristic	Children (17 cases)	Adults (73 cases)	*P-*value
	No. (%)	No. (%)	
Sex			0.99
Male	7 (41)	30 (41)	
Female	10 (59)	43 (59)	
Symptom			<0.01
Symptomless	4 (19)	38 (49)	
Haematuria	8 (38)	22 (28)	
Abdominal mass	5 (24)	3 (4)	
Flank pain	4 (19)	15 (19)	
Laterality			0.61
Left	7 (41)	35 (47)	
Right	10 (59)	38 (53)	
Operation			0.03
RN	16 (94)	49 (67)	
NSS	1 (6)	24 (33)	
pT stage			0.99
T1/T2	13 (76)	54 (74)	
T3/T4	4 (24)	19 (26)	
Nuclear grade			0.49
1/2	12 (71)	45 (62)	
3/4	5 (29)	28 (38)	
AJCC stage			<0.01
I/II	5 (29)	49 (67)	
III/IV	12 (71)	24 (33)	
Local lymph node metastasis			0.02
LN+	10 (59)	21 (29)	
LN−	7 (41)	52 (71)	
Vena cava tumour thrombosis			0.68
Positive	1 (6)	9 (12)	
Negative	16 (94)	64 (88)	
Adjuvant therapy			<0.01
None	14 (82)	23 (31)	
Immunotherapy	2 (12)	31 (43)	
Targeted therapy	1 (6)	19 (26)	
Tumour boundary			0.08
Clear	6 (35)	43 (59)	
Unclear	11 (65)	30 (41)	

RN, Radical nephrectomy; NSS, Nephron-sparing surgery; AJCC, American Joint Committee On Cancer; LN+, Lymph node positivity; LN−, Lymph node negativity.

### The outcomes of follow-up

Except for five patients who were lost to follow-up, the remaining cases were followed up, with a mean duration of 52 months (range: 1–137 months) for the children and 46 months (range: 4–129 months) for the adults. only one adult patient (1.1%) had sarcomatoid tissue in the tumour and died of multiple organ metastasis 24 months after surgery. Of 10 adult patients (11.1%) with coagulative tumour necrosis, 3 cases had disease progression and adverse outcome, and the remaining 7 cases stayed stable. Four cases of adult patients with pT1/pT2 and LN+ were followed up, and 3 cases had an adverse outcome. Of 3 children with disease progression, only one child died of brain metastasis 46 months after the operation, and the remaining 2 recurrence cases had local LN+, followed by the dissection of local lymph nodes. Of the 24 adults with disease progression, 16 cases died of distant metastasis with a mean follow-up time of 30 months: 8 cases in a distant lymph node, 3 cases in the liver, 2 cases in the lung, 1 case in the brain, 1 case in the bone, 1 case in the peritoneum, and the other patients were stable. Survival analysis showed that pT stage, adjuvant treatment, local lymph node metastasis, AJCC stage and vena cava tumour thrombosis influenced OS (all *P* < 0.05) and that tumour boundary, pT stage, adjuvant treatment, local lymph node metastasis, AJCC stage and vena cava tumour thrombosis were associated with PFS (all *P* < 0.05) in the adult group (Table 3). Furthermore, adjuvant therapy and pT stage were associated with PFS (all *P* < 0.05) in the paediatric group (Table 4). Multivariable analysis was executed to estimate the prognostic value of the clinicopathologic characteristics for PFS and OS. Due to the small sample size and favourable prognosis of the children, multivariable analysis was conducted only in the adults. When performing multivariable analysis, both adjuvant treatment and AJCC stage were excluded to obtain a more reliable result because they were closely associated with postoperative pathological stage. Multivariable analysis indicated that local lymph node metastasis was associated with both PFS (HR = 0.10; 95% CI 0.02–0.52; *P* = 0.01) and OS (HR = 0.11; 95% CI 0.01–0.98; *P* = 0.04) in adults (Table 5).

**Table 3:** Survival analysis of clinicopathologic variables for overall survival (OS) and progression-free survival (PFS) in the adult group.

Variable	One-year	Five-year	One-year	Five-year	*P*-value	
	OS	OS	PFS	PFS	Log-rank test	
	% (SE)	% (SE)	% (SE)	% (SE)	OS	PFS
Sex					0.29	0.17
Male	89.4 (0.06)	71.3 (0.10)	69.2 (0.09)	58.0 (0.09)		
Female	100	78.5 (0.07)	85.5 (0.06)	67.3 (0.08)		
Symptom					—^*^	—^*^
Symptomless	97.3 (0.03)	78.5 (0.08)	83.9 (0.06)	68.2 (0.08)		
Haematuria	90.0 (0.07)	78.5 (0.10)	72.0 (0.10)	59.7 (0.11)		
Abdominal mass	—^*^	—	—	—		
Flank pain	100	51.4 (0.19)	72.7 (0.13)	51.9 (0.16)		
Laterality					0.80	0.78
Left	93.6 (0.04)	71.8 (0.10)	78.5 (0.07)	67.9 (0.09)		
Right	97.3 (0.03)	78.2 (0.07)	78.9 (0.07)	60.2 (0.09)		
Operation					0.78	0.16
RN	95.6 (0.03)	75.9 (0.07)	74.3 (0.06)	57.5 (0.08)		
NSS	95.8 (0.04)	72.6 (0.13)	87.5 (0.07)	75.4 (0.10)		
pT stage					<0.01	<0.01
T1/T2	100	90.0 (0.05)	92.4 (0.04)	82.4 (0.06)		
T3/T4	82.4 (0.09)	34.0 (0.13)	36.7 (0.12)	0		
Nuclear grade					0.64	0.50
1/2	95.3 (0.03)	75.6 (0.07)	79.7 (0.06)	67.1 (0.07)		
3/4	96.0 (0.04)	74.6 (0.10)	77.2 (0.08)	53.6 (0.13)		
AJCC stage					<0.01	<0.01
I/II	100	91.9 (0.05)	93.7 (0.04)	88.9 (0.05)		
III/IV	85.7 (0.08)	38.5 (0.12)	46.6 (0.11)	0		
Local lymph node metastasis					<0.01	<0.01
LN+	84.2 (0.08)	40.3 (0.13)	40.0 (0.11)	0		
LN−	100	89.1 (0.05)	93.9 (0.03)	87.3 (0.05)		
Vena cava tumour thrombosis					<0.01	<0.01
Positive	88.9 (0.11)	22.2 (0.14)	11.1 (0.11)	0		
Negative	96.6 (0.02)	85.1 (0.05)	88.6 (0.04)	72.8 (0.06)		
Adjuvant therapy					<0.01	<0.01
None	95.7 (0.04)	87.7 (0.09)	95.7 (0.04)	85.2 (0.08)		
Immunotherapy	100	90.5 (0.07)	86.4 (0.06)	82.7 (0.07)		
Targeted therapy	88.9 (0.07)	43.8 (0.12)	44.4 (0.11)	11.1 (0.07)		
Tumour boundary					0.35	0.02
Clear	97.6 (0.02)	80.0 (0.08)	88.0 (0.05)	71.1 (0.08)		
Unclear	92.6 (0.05)	68.6 (0.09)	65.2 (0.09)	50.5 (0.10)		

RN, Radical nephrectomy; NSS, Nephron-sparing surgery; AJCC, American Joint Committee On Cancer; LN+, Lymph node positivity; LN−, Lymph node negativity; SE, Standard error; *No data or insufficient data for statistical analysis.

**Table 4:** Survival analysis of clinicopathologic variables for overall survival (OS) and progression-free survival (PFS) in the paediatric group.

Variable	One-year	Five-year	One-year	Five-year	*P*-value	
	OS	OS	PFS	PFS	Log-rank test	
	% (SE)	% (SE)	% (SE)	% (SE)	OS	PFS
Sex					0.32	0.36
Male	100	66.7 (0.27)	60 (0.22)	60 (0.22)		
Female	100	100	85.7 (0.13)	85.7 (0.13)		
Symptom					—^*^	—^*^
Symptomless	100	—	100	—		
Haematuria	100	75.0 (0.22)	80 (0.18)	80 (0.18)		
Abdominal mass	—^*^	—	—	—		
Flank pain	100	—	100	—		
Laterality					0.16	0.13
Left	100	100	100	100		
Right	100	50.0 (0.35)	57.1 (0.19)	57.1 (0.19)		
Operation					—	—
RN	100	83.3 (0.15)	74.1 (0.13)	74.1 (0.13)		
NSS	—	—	—	—		
pT stage					0.32	0.24
T1/T2	100	100	87.5 (0.12)	87.5 (0.12)		
T3/T4	100	66.7 (0.27)	50.0 (0.25)	50.0 (0.25)		
Nuclear grade					0.48	0.73
1/2	100	75.0 (0.22)	76.2 (0.15)	76.2 (0.15)		
3/4	100	100	66.7 (0.27)	66.7 (0.27)		
AJCC stage					0.48	0.27
I/II	100	100	100	100		
III/IV	100	75.0 (0.22)	64.8 (0.17)	64.8 (0.17)		
Local lymph node metastasis					0.48	0.27
LN+	100	75.0 (0.22)	64.8 (0.17)	64.8 (0.17)		
LN−	100	100	100	100		
Vena cava tumour thrombosis					0.66	0.58
Positive	100	100	100	100		
Negative	100	80.0 (0.18)	71.6 (0.14)	71.6 (0.14)		
Adjuvant therapy					—	—
None	100	83.3 (0.15)	88.9 (0.11)	88.9 (0.11)		
Immunotherapy	—	—	—	—		
Targeted therapy	—	—	—	—		
Tumour boundary					0.32	0.21
Clear	100	100	100	100		
Unclear	100	66.7 (0.27)	62.5 (0.17)	62.5 (0.17)		

RN, Radical nephrectomy; NSS, Nephron-sparing surgery; AJCC, American Joint Committee On Cancer; LN+, Lymph node positivity; LN−, Lymph node negativity; SE, Standard error; *No data or insufficient data for statistical analysis.

**Table 5:** Multivariable analysis of overall survival and progression-free survival in adult patients.

Variable	Progression-free survival				Overall survival			
	HR	95% CI		*P*-value	HR	95% CI		*P*-value
		Lower	Upper			Lower	Upper	
Sex	1.90	0.66	5.47	0.24	1.48	0.44	4.99	0.52
Operation	1.90	0.58	5.40	0.32	0.99	0.26	3.78	0.99
Nuclear grade	0.80	0.28	2.29	0.67	0.67	0.17	2.63	0.57
pT stage	1.55	0.30	7.99	0.60	1.04	0.12	9.08	0.97
Local lymph node metastasis	0.10	0.02	0.51	0.01	0.11	0.01	0.98	0.04
Vena cava tumour thrombosis	0.43	0.13	1.43	0.17	0.52	0.11	2.44	0.41
Tumour boundary	1.45	0.56	3.79	0.45	0.78	0.25	2.46	0.68

CI, Confidence interval; HR, Hazard ratio.

## Discussion

Xp11 translocation RCCs were classified as MiT family translocation carcinomas with an incidence among diagnosed RCCs of one-third in paediatric patients and approximately 0.2–5.0% in adults^8,9,15^. The incidence of adults in previous studies^7,16^ was broadly consistent with ours, while the incidence of children was significantly underestimated in our multi-centre study, which could be caused by insufficient sample size. Although children diagnosed with Xp11 translocation RCCs exhibit a high incidence compared to adults, adult Xp11 translocation RCCs could still outnumber paediatric Xp11 translocation RCCs in that RCCs are more common in adults. In addition, our study inferred that the incidence of female Xp11 translocation RCCs in the adult group was significantly higher than that in the male group, as it is a rare malignant tumour in the urinary system with a higher incidence in females. Meta-analysis confirmed that females were more predominant than males in adult patients, while in children, no sex-related predominance was observed^13^. However, further exploration is still essential to understand why there is an age-related difference in incidence and a high incidence in adult female patients.

The clinical manifestations of Xp11 translocation RCCs are basically similar to other types of RCCs. Statistical results indicate that haematuria is the most common symptom in the Xp11 translocation RCCs, which is consistent with the findings of several clinical studies^8,17^. Over the past decades, the methods of diagnosing Xp11 translocation RCCs have been gradually appreciated and deeply studied. Recent studies have confirmed that TFE3-IHC combined with the FISH assay is an effective method for the diagnosis of Xp11 translocation RCCs, which could improve the specificity and may reduce false-positive results^6,16^. Therefore, all eligible patients were diagnosed by TFE3-IHC combined with the TFE3 break-apart FISH assay in the present research. Interestingly, it has been confirmed that dual-fusion FISH probes were useful for identifying ASPL-TFE3 and PRCC-TFE3 RCC^18,19^, and other probes are worthy of designing to diagnose the remaining known subtypes of Xp11 translocation RCCs.

Considering that Xp11 translocation RCCs are obviously more invasive and prone to lymph node and distant metastasis in adults, taking the most appropriate clinical therapy is of great significance. To date, although there is no agreement on clinical treatments for Xp11 translocation RCCs, the recommendation for all surgeries is RN if possible. As shown in our study, RN was performed on the majority of patients, especially in the paediatric group. We then inferred from the statistical results that 10 of 13 cases in the paediatric group who received RN showed no evidence of progression with a mean follow-up time of 57 months. Similarly, good outcomes have been reported in 8 of 9 cases of Xp11 translocation RCCs, which were confirmed by comparative genomic hybridization 3–10 years after RN^20^. However, with the development of laparoscopic technology and the application of the Da Vinci robotic surgery system, NSS is popularly recommended in solitary renal tumours with diameters of less than 4 cm^21^. Cheng *et al*. found that treating small Xp11 translocation RCCs (≤4 cm) with intact pseudocapsules by receiving NSS produced a favourable treatment outcome, which provided a theoretical basis for NSS treatment^22^. In addition, Gorin *et al*. reported that 4 patients with T1a Xp11 translocation RCCs who received partial nephrectomy for an incidentally detected small renal mass (mean imaging diameter: 2.6 cm) were alive without evidence of disease during a mean follow-up of 37 months^23^. Moreover, Liu *et al*. reported that 9 children with Xp11 translocation RCCs who underwent NSS were alive without postoperative recurrence and metastasis for the mean follow-up period of 50.1 months and concluded that NSS is safe and feasible for Xp11 translocation RCCs sized < 4–7 cm in diameter and located in one pole^24^. Our statistical results implied that NSS was performed for the treatment of 24 Xp11 translocation RCCs, and most patients had a favourable prognosis. Although many recent studies and guidelines have extended NSS to RCCs, most of these subjects are CCRCC or other common subtypes of RCC^21,25^. Considering that Xp11 translocation RCCs are more aggressive, more clinical studies with large sample sizes in multiple centres are needed to determine whether NSS is feasible and safe for Xp11 translocation RCCs.

Compared with traditional types of RCC, patients with Xp11 translocation RCCs often presented at an advanced stage and demonstrated an invasive clinical course and poor prognosis^10^. Moreover, the clinical behaviour of Xp11 translocation RCCs that occurred in adults was more aggressive^26^. However, Song *et al*. reported that progression-free survival was observed in 13 of 15 children after surgery and concluded that paediatric patients with LN+ had a good prognosis following surgery alone^27^. The results from our research confirmed that the prognosis of adults in higher AJCC stage was poorer compared with that of children. A recent clinical study suggested that cancer-specific survival differed significantly between adult patients with positive TFE3 rearrangement RCCs and adult patients with negative TFE3 rearrangement PRCC, which implied that adult RCCs with TFE3 rearrangement may be a clinically aggressive tumour^9^. It has been presented in our study that LN+ is associated with an increased risk of disease progression and death in adults. Therefore, adult patients diagnosed with Xp11 translocation RCCs with higher AJCC stage and LN+ need more active follow-up and treatments after surgery. The significant difference in the prognosis between children and adults deserves our in-depth study. Basic research may be able to find a reasonable explanation at the genetic level.

There are some limitations to our study. First, although our study is the first multi-institutional clinical design in China concerning Xp11 translocation RCCs, the sample size was still not sufficiently large due to the low incidence of this rare disease and potential missed diagnosis, and the follow-up time for patients receiving NSS was relatively short. Long-term follow-up is essential to estimating the therapeutic efficacy of NSS. Second, the multi-institutional study is advantageous for reducing biases caused by single centre studies, while it is also subject to heterogeneity in data collection and follow-up. Third, we have to note that we did not finish identifying the gene-fusion partners for all patients, and further study on genetic fusion types using fusion probes is in progress in our centres.

## Conclusions

We have demonstrated that Xp11 translocation RCCs are rare entities of RCC and differ in age-related incidence. The results from survival analysis suggest that adult patients with LN+ have higher progression and poorer prognosis compared with those of children. Local lymph node metastasis could be associated with an increased risk of disease progression and death outcome in adults, and adult patients diagnosed with Xp11 translocation RCCs with higher AJCC stage and LN+ need more active follow-up and treatments after surgery.

## Supplementary information

Supplementary information

## Data Availability

The datasets generated during and/or analysed during the current study are available from the corresponding author on reasonable request.
