# Supplementary material for: Comparative Clinicopathologic Characteristics and Outcomes of Paediatric and Adult Xp11 Translocation Renal Cell Carcinomas: a Retrospective Multicentre Study in China
Source: Sci Rep. 2020 Feb 10;10:2249. doi: 10.1038/s41598-020-59162-5 (PMC7010786; doi:10.1038/s41598-020-59162-5)
Supplement: Supplementary file 1 — Supplementary information [file 41598_2020_59162_MOESM1_ESM.pdf]

Comparative Clinicopathologic Characteristics and Outcomes of Paediatric and Adult Xp11 Translocation Renal Cell Carcinomas: a Retrospective Multicentre Study in China

Authors: Wenliang Ma[1], Ning Liu[2], Wenyuan Zhuang[2], Weijian Li[2], Feng Qu[2], Jing Sun[3], Wei Xu[4], Lihua Zhang[5], Ruipeng Jia[6], Linfeng Xu[2], Xiaozhi Zhao[2], Xiaogong Li[2], Gutian Zhang[2], Hongqian Guo[2], Dongmei Li[7,8], Weidong Gan[1,2*]

Institutions: [1]Department of Urology, Drum Tower Clinical Medical School of Nanjing Medical University, Nanjing, Jiangsu, China;

[2]Department of Urology, Nanjing Drum Tower Hospital, The Affiliated Hospital of Nanjing University Medical School, Nanjing, Jiangsu, China;

[3]Department of Oncology, Jiangsu Province Hospital, The First Affiliated Hospital of Nanjing Medical University, Nanjing, Jiangsu, China;

[4]Department of Pathology, Jiangsu Cancer Hospital, The Affiliated Cancer Hospital of Nanjing Medical University, Nanjing, Jiangsu, China;

[5]Department of Pathology, Zhongda Hospital Southeast University, Nanjing, Jiangsu, China;

[6]Department of Urology, Nanjing First Hospital, The Affiliated Nanjing Hospital of Nanjing Medical University, Nanjing, Jiangsu, China;

[7]Immunology and Reproduction Biology Laboratory & State Key Laboratory of Analytical Chemistry for Life Science, Medical School, Nanjing University, Nanjing, Jiangsu, China;

[8]Jiangsu Key Laboratory of Molecular Medicine, Nanjing University, Nanjing, Jiangsu, China

Corresponding author: Weidong Gan, Nanjing Drum Tower Hospital, No. 321 Zhongshan Road, Nanjing, Jiangsu Province, People's Republic of China. 210008. Email: gwd@nju.edu.cn. Tel: +86 13305186699; Fax: +86 2583307115

Supplementary table S1 Survival analysis based on institutions for overall survival (OS) and progression-free survival (PFS) in 85 patients. SE, Standard error; *No data or insufficient data for statistical analysis.

Variable	One-year OS % (SE)	Five-year OS % (SE)	One-year PFS % (SE)	Five-year PFS % (SE)	P-value Log-rank test OS	P-value Log-rank test PFS
Institutions					0.24	0.40
Nanjing Drum Tower Hospital	97.9 (0.02)	77.9 (0.07)	76.1 (0.06)	66.2 (0.07)		
Jiangsu Province Hospital	93.3 (0.06)	73.8 (0.14)	80.0 (0.10)	58.3 (0.13)		
Jiangsu Cancer Hospital	85.7 (0.13)	35.7 (0.27)	71.4 (0.17)	42.9 (0.19)		
Zhongda Hospital Southeast University	100	-*	85.7 (0.13)	85.7 (0.13)		
Nanjing First Hospital	100	-	100	-		
Age					0.29	0.57
Children (<18 years)	100	83.3 (0.15)	74.1 (0.13)	74.1 (0.13)		
Adults (≥18 years)	95.6 (0.03)	75.5 (0.06)	80.0 (0.05)	64.0 (0.06)		

Supplementary table S2 Survival analysis based on institutions for overall survival (OS) and progression-free survival (PFS) in adult group. SE, Standard error; *No data or insufficient data for statistical analysis.

Variable	One-year OS % (SE)	Five-year OS % (SE)	One-year PFS % (SE)	Five-year PFS % (SE)	P-value Log-rank test OS	P-value Log-rank test PFS
Institutions					0.40	0.45
Nanjing Drum Tower Hospital	97.6 (0.02)	78.1 (0.07)	75.2 (0.07)	63.7 (0.08)		
Jiangsu Province Hospital	92.9 (0.07)	73.0 (0.14)	85.7 (0.09)	62.5 (0.14)		
Jiangsu Cancer Hospital	85.7 (0.13)	35.7 (0.27)	71.4 (0.17)	42.9 (0.19)		
Zhongda Hospital Southeast University	100	-*	100	-		
Nanjing First Hospital	100	-	100	-		

Supplementary table S3 Multivariable analysis of overall survival and progression-free survival in 85 patients. CI, Confidence interval; HR, Hazard ratio.

Variable	Progression-free survival				Overall survival			
	HR	95% CI		*P*-value	HR	95% CI		*P*-value
		Lower	Upper			Lower	Upper	
Sex	1.52	0.64	3.60	0.34	1.22	0.41	3.66	0.72
Operation	1.16	0.40	3.40	0.78	0.69	0.18	2.62	0.59
Nuclear grade	0.77	0.33	1.82	0.55	0.34	0.09	1.31	0.12
pT stage	0.29	0.07	1.16	0.08	0.81	0.11	5.96	0.84
Local lymph node metastasis	3.78	1.02	14.04	0.04	0.10	0.01	0.80	0.03
Vena cava tumour thrombosis	0.64	0.25	1.69	0.37	0.33	0.08	1.35	0.12
Tumour boundary	1.26	0.51	3.14	0.62	0.82	0.25	2.71	0.74
Institutions	1.04	0.78	1.40	0.79	0.28	0.07	1.18	0.08
